# Sirtuins: from metabolic regulation to brain aging

**DOI:** 10.3389/fnagi.2013.00036

**Published:** 2013-07-23

**Authors:** Wenzhen Duan

**Affiliations:** ^1^Division of Neurobiology, Department of Psychiatry and Behavioral Sciences, Johns Hopkins University School of MedicineBaltimore, MD, USA; ^2^Department of Neuroscience, Johns Hopkins University School of MedicineBaltimore, MD, USA; ^3^Program in Cellular and Molecular Medicine, Johns Hopkins University School of MedicineBaltimore, MD, USA

**Keywords:** SIRT1, sirt3, mitochondrial metabolism, brain aging, calorie restriction

## Abstract

Brain aging is characterized by progressive loss of neurophysiological functions that is often accompanied by age-associated neurodegeneration. Calorie restriction has been linked to extension of lifespan and reduction of the risk of neurodegenerative diseases in experimental model systems. Several signaling pathways have been indicated to underlie the beneficial effects of calorie restriction, among which the sirtuin family has been suggested to play a central role. In mammals, it has been established that sirtuins regulate physiological responses to metabolism and stress, two key factors that affect the process of aging. Sirtuins represent a promising new class of conserved deacetylases that play an important role in regulating metabolism and aging. This review focuses on current understanding of the relation between metabolic pathways involving sirtuins and the brain aging process, with focus on SIRT1 and SIRT3. Identification of therapeutic agents capable of modulating the expression and/or activity of sirtuins is expected to provide promising strategies for ameliorating neurodegeneration. Future investigations regarding the concerted interplay of the different sirtuins will help us understand more about the aging process, and potentially lead to the development of therapeutic approaches for the treatment of age-related neurodegenerative diseases and promotion of successful aging.

## INTRODUCTION

The brain, similar to other organs, undergoes a gradual decline in energy metabolism during aging (Drew and Leeuwenburgh, 2004; Navarro and Boveris, 2007; Boveris and Navarro, 2008; Swerdlow, 2011). Since neurons require large amounts of energy for the firing of action potential, neurotransmission, and other processes, the age-related decline in metabolism contributes to the cognitive declines associated with aging (Biessels and Kappelle, 2005; Boveris and Navarro, 2008). Aging is also a risk factor for age-associated diseases such as neurodegenerative disorders. These diseases may occur when neurons fail to respond adaptively to an age-related decline in basal metabolic rates and in energy-driven tasks, such as neuromuscular coordination, cognitive performance, and environmental awareness (Swerdlow, 2007). In the past decade, the function of mammalian sirtuins, evolutionarily conserved nicotinamide adenine dinucleotide (NAD)-dependent protein deacetylases/ADP-ribosyltransferases, has been investigated in greater detail, and we now have a much better molecular understanding of the multiple roles that this unique family of enzymes plays in aging and seemingly every biological process. There is little doubt that sirtuins have emerged as critical modulators of metabolic adaptive responses, and their activities have been linked to multiple diseases, from metabolic abnormalities to neurodegeneration.

Sirtuins were originally identified as one of the genes that regulate the mating types of budding yeast, *Saccharomyces cerevisiae*, and named silent information regulator 2 (Sir2) in lower organisms (Klar and Fogel, 1979). Following the first publication describing a role for yeast Sir2 in promoting longevity (Kaeberlein et al., 1999), many investigations focused on elucidating whether sirtuins might play similar roles in other organisms. Sirtuins have been shown to regulate lifespan in lower organisms, including yeast, nematodes, and fruit flies (Haigis and Guarente, 2006), although their role in worm and fly lifespan has recently been debated (Burnett et al., 2011; Viswanathan and Guarente, 2011). Most of these studies have described a key role for SIRT1 in regulating the metabolic response to calorie restriction (CR; Canto and Auwerx, 2009), a dietary intervention that robustly extends life span across numerous species. However, whole body overexpression of SIRT1 in mice does not affect life span (Herranz et al., 2010). Nevertheless, SIRT1 does appear to promote healthy aging by protecting against several age-related pathologies, such as facilitating insulin sensitivity, elevating glucose production, reducing oxidative stress, potentiating activity of brain-derived neurotrophic factor (BDNF) transcriptional factor cAMP response element-binding protein (CREB; Guarente and Franklin, 2011).

Mammals have seven sirtuins (SIRT1–7) which are found in different subcellular locations, including the nucleus (SIRT1, SIRT6, and SIRT7), cytosol (SIRT2), and mitochondria (SIRT3, SIRT4, and SIRT5). Most of the studies have described a key role for SIRT1 in regulating the metabolic response to CR (Canto and Auwerx, 2009), a dietary regimen involving reduced 30–40% calorie intake compared to normal calorie intake, that resulted in extended lifespan and reduced development of morbidity with aging (Jiang et al., 2000; Masoro, 2000; Sinclair, 2002; Koubova and Guarente, 2003). Calorie restriction is the only intervention that has consistently been shown to delay the onset, slow the progression of age-related disease, and extend lifespan in short-lived species, as well as in long-lived non-human primates, suggesting that similar mechanisms would be operative in humans. Whole body overexpression of Sirt1 in mice does not affect lifespan (Herranz et al., 2010). Nevertheless, SIRT1 promotes healthy aging by preventing age-associated pathologies (Guarente and Franklin, 2011). Another strong link between mammalian sirtuins and the anti-aging effects of CR was provided by SIRT3, which mediates the prevention of age-related hearing loss (Someya et al., 2010). SIRT3 is required for the CR-mediated reduction of oxidative damage in multiple tissues via regulation of the glutathione antioxidant system (Someya et al., 2010).

In this review, we focus on the effects of SIRT1 and SIRT3 on metabolic regulation and their anti-aging activity in brain, and further discuss potential pharmacological approaches to remedy and prevent age-associated neurological disorders by targeting sirtuins.

## SIRT1, METABOLISM AND BRAIN AGING

### DISTRIBUTION OF SIRT1 IN THE BRAIN

During mouse embryogenesis, SIRT1 is highly expressed in the brain, spinal cord, and dorsal root ganglion, with the peak expression at E4.5 (Salminen and Kaarniranta, 2012). SIRT1 is also expressed in the adult brain, with high levels in the cortex, hippocampus, cerebellum, and hypothalamus, and low levels in white matter (Singh, 2004). Among the various cell types of brain, SIRT1 is predominantly, if not exclusively, expressed in neurons (Singh, 2004; Adler et al., 2007; Salminen and Kaarniranta, 2012). The only exception is that SIRT1 is found in microglia when co-cultured with neurons (Schmitz et al., 2004). At the subcellular level, SIRT1 is viewed as a nuclear protein (Chen and Greene, 2003). Yet it is reported that SIRT1 has both nuclear import and export sequences, and that SIRT1 is present in the cytosolic fraction of mouse brain, although its cytosolic function is just beginning to be elucidated (Chen et al., 2005b; Lee et al., 2008; Hardie, 2011).

### SIRT1 MEDIATES METABOLIC BENEFITS UNDER CR

SIRT1 contains 747 amino acids in humans, with a predicted molecular weight of 81 kDa and a measured one of 120 kDa. In addition to histones, SIRT1 also deacetylates a number of non-histone substrates, including p53 (Luo et al., 2001)and peroxisome proliferator-activated receptor γ (PPARγ) coactivator-1α (PGC-1α; Nemoto et al., 2005), and FOXO (Xiong et al., 2011), nuclear factor κ-light-chain-enhancer of activated B cells (NF-κB; Salminen et al., 2008b). SIRT1 is drawing even more attention since it is considered to be one of the determining factors in lifespan extension induced by CR, a phenomenon observed in phylogenetically diverse organisms including yeast, worm, fruit fly, and mouse (Kaeberlein et al., 1999; Tissenbaum and Guarente, 2001; Howitz et al., 2003; Rogina and Helfand, 2004). Its beneficial roles are further supported by the findings that putative SIRT1-activating compounds, such as resveratrol, also promote longevity in several species, including yeast (Howitz et al., 2003), worm (Wood et al., 2004), and mouse (Baur et al., 2006), making it an anti-aging target.

**FIGURE 1:**
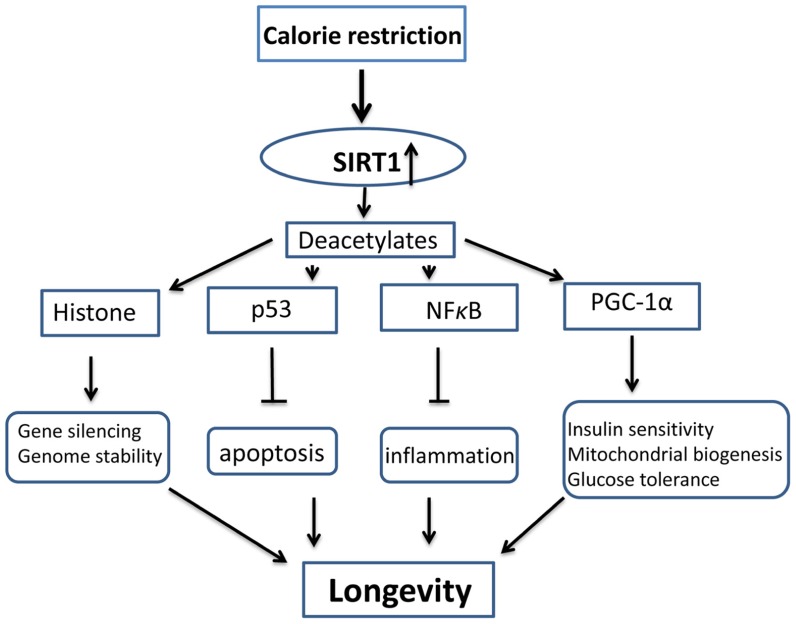
** Schematic diagram of anti-aging mechanism activated by SIRT1 and calorie restriction.** Calorie restriction upregulates the activity and levels of SIRT1, SIRT1 deacetylates its substrates, including histone and non-histone molecules, and improve genome stability, prevent apoptosis and inflammation, and increases mitochondrial biogenesis, insulin sensitivity and glucose tolerance. All these effects result in longevity.

The effects of SIRT1 on longevity rely on its enzymatic activity of deacetylation of histone and non-histone substrates. While the deacetylation of histones leads to their interaction with DNA and consequent gene silencing (Braunstein et al., 1993; Sauve et al., 2006; Dali-Youcef et al., 2007), the deacetylation of non-histone proteins has a wide range of biological effects, including metabolic adjustment, survival promotion, and autophagy (Campisi, 2005; Dali-Youcef et al., 2007; Brooks and Gu, 2009; Madeo et al., 2010). For example, SIRT1 inhibits p53 (Luo et al., 2001), reducing its pro-apoptotic effect. It also inhibits NF-κB (Yeung et al., 2004), reducing its pro-inflammatory effects. In contrast, SIRT1 activates a transcriptional coactivator, PGC-1α (Nemoto et al., 2005), leading to increased glucose levels, insulin sensitivity, and mitochondrial biogenesis. Together, these and other effects, contribute to the longevity evoked by CR (**Figure 1**).

These metabolic changes and the cytoprotective involvement of CR are generally considered to occur in non-neural organs, such as the liver, pancreas, muscle, and fat tissues (Brooks and Gu, 2009; Imai and Guarente, 2010). However, recent studies suggest that the hypothalamus may also contribute to the longevity effects of SIRT1 and CR via coordination of neurobehavioral and neuroendocrine changes, including body temperature, appetite, and overall physical activity (Dietrich et al., 2010; Satoh et al., 2010). SIRT1 is abundantly expressed in several regions in the hypothalamus of mice, especially in the arcuate, paraventricular, ventro- and dorsomedial nuclei; and CR increases SIRT1 levels in the hypothalamus, which increases body temperature, food intake, and physical activity (Ramadori et al., 2008; Dietrich et al., 2010; Satoh et al., 2010). SIRT1 appears to be required for the aforementioned behavioral changes, which are prevented if SIRT1 is knocked out or inhibited (Chen et al., 2005a; Satoh et al., 2010). In addition to the hypothalamus, SIRT1 is also expressed in other regions of the brain, including the cortex, striatum, and hippocampus (Ramadori et al., 2008). Shortly after this finding, a neuroprotective role of SIRT1, has been reported (Tang, 2009; Morris et al., 2011).

### SIRT1 AND FACTORS INVOLVED IN CR AND AGING

SIRT1 has been found to delay aging and promote longevity by regulating the activity of key cellular proteins like p53, FOXO and Ku70 that are involved in either apoptotic processes or cellular repair mechanisms. SIRT1 may thus promote health and longevity partly by either decelerating cell death and/or by boosting repair mechanisms in the cells (Wang et al., 2010).

It has become increasingly evident that the salutary effects of the CR, are in part due to the promotion of sirtuins (Wang et al., 2010). The expression levels of SIRT1 increase upon CR in several rodent and human tissues, including white adipose, liver, skeletal muscle, brain, and kidney. Levels of NAD have been shown to rise in liver cells under CR-like conditions, which in turn induces expression of SIRT1 (Rodgers et al., 2005). SIRT1 ends up consuming NAD+ as a result of its deacetylase activity, generating nicotinamide, an inhibitor of its own activity. NAD+ is known to protect neurons (Liu et al., 2009) and thus by increasing the levels of NAD+, CR may preserveSIRT1 activity. SIRT1 also activates PGC1α (Rodgers et al., 2005) which results in mitochondrial biogenesis (Liu et al., 2009). A decline in mitochondrial activity is thought to be causative in many age-related diseases (Petersen et al., 2003; Singh, 2004). CR evokes improvements in mitochondrial activity similar to those of SIRT1. Therefore, it is possible that small-molecule modulators of SIRT1 may act on the same pathways as those modified by CR, and thus have potential to mitigate age-related diseases (Lavu et al., 2008).

SIRT1 interacts with and modulates other key factors involved in mammalian aging, such as NF-κB that controls a low-grade systemic inflammation along with human aging process-inflamm-aging (Salminen et al., 2008a), mammalian target of rapamycin (mTOR; Finley and Haigis, 2009), AMP-activated protein kinase (AMPK; Salminen and Kaarniranta, 2012), therefore controls the gin process. The aging process involves changes in immune regulation; NF-κB signaling is the master regulator of the immune system. Inhibition of NF-κB signaling in aged mice reverted the tissue characteristics and global gene expression to those of young mice (Adler et al., 2007). The function of the NFκB complex can be regulated by the acetylation of the p65 component (Schmitz et al., 2004). SIRT1 can interact with RelA/p65 protein in the NF-κB complex and specifically deacetylates lysine 310, which has been demonstrated to potentiate the transactivation capacity of the NFκB complex (Chen and Greene, 2003). Several studies have indicated that SIRT1 is a potent inhibitor of NF-κB transcription (Yeung et al., 2004; Chen et al., 2005b). The signaling link between SIRT1 and NF-κB is especially interesting with respect to aging, as a consequence of the release of the SIRT1 brake, the transactivation efficiency of NF-κB factor is potentiated, which evokes immune activation and inflamm-aging.

Aging process is also regulated by autophagy. It has been identified the signaling pathways that regulate autophagic degradation and SIRT1 is a potent regulator of autophagic degradation (Lee et al., 2008), SIRT1 can interact with and deacetylates several components in the complexes of forming autophagosomes, such as Atg5, Atg7, and Atg8 proteins (Lee et al., 2008). There is a clear overlap between the signaling networks regulating both aging and autophagocytosis, which emphasizes the important role of autophagy in the regulating of aging and age-related degenerative diseases. It is evident that increase in autophagy can extend lifespan. mTOR activity is suppressed by CR, reduction in mTOR signaling is a logic candidate mechanism for the anti-aging benefits of CR. Through deacetylation of a variety of proteins involved in autophagy process, SIRT1 can regulate physiological process during aging and moderated by CR (Haigis and Guarente, 2006).

Efficient control of energy metabolic homeostasis is a hallmark of improved healthspan and extended lifespan. The AMPK and SIRT1 signaling pathways are highly conserved energy sensor of increased levels of AMP and NAD^+^, respectively, AMPK signaling is involved in the regulation of energy metabolic homeostasis (Hardie, 2011). Canto and Auwerx (2009) demonstrated that the activation of AMPK stimulated the functional activity of SIRT1 by increasing the intracellular concentration of NAD^+^. Interestingly, SIRT1 was able to deacetylate LKB1 kinase which subsequently increased its activity (Lan et al., 2008). Since LKB1 is an upstream activator of AMPK, this signaling pathway stimulates the activation of AMPK. This positive feedback loop between SIRT1 and AMPK can also potentiate the function of the other AMPK-activated signaling pathways. The close relationship between AMPK and SIRT1 is evidence that energy balance effectively controls cellular responses via an integrated signaling network. AMPK can inhibit the activity of mTOR complex via two different mechanisms, either by directly phosphorylating the Raptor, a regulatory component of mTORC1, or by the phosphorylation of tuberous sclerosis protein 2 (TSC2), which subsequently suppresses the activity of mTOR (Jung et al., 2010; Mihaylova and Shaw, 2011). Taken together, SIRT1 interacts with other key anti-aging signaling pathways thereby contributing to longevity control.

It has been established that aging is a known risk factor for many neurodegenerative diseases including Alzheimer’s disease (AD), Parkinson’s disease (PD), Wallerian neurodegeneration, Huntington’s disease (HD), and amyotrophic lateral sclerosis (ALS). The pathomechanisms involved in these disorders involve common biochemical pathways and processes, including protein misfolding, oligomerization, and aggregation, proteolysis, post-translational modifications, mitochondrial dysfunction, abnormal metabolic processes, and proinflammatory and proapoptotic responses that we discuss in the next section.

### SIRT1 AND AGE-ASSOCIATED NEUROLOGICAL DISEASES

#### Wallerian degeneration

Wallerian degeneration refers to axonal death and degradation after focal injury, followed by breakdown of myelin sheath. The neuroprotective effect of SIRT1 against Wallerian degeneration was first discovered in wlds transgenic mice (Perry et al., 1990). These mice exhibited a significant delay in axonal degeneration after physical or chemical injury. The mechanistic basis for the delayed axonal damage was apparently associated with the mutant wlds chimeric protein. It has been shown that Nicotinamide mononucleotide adenylyltransferase 1 (NMNAT-1) activity plays an important role in the prevention of axonal damage, exerting its protective effects through SIRT1 activation, as the neuroprotection is blocked by the SIRT1 inhibitor sirtinol or siRNA-mediated SIRT1 silencing (Araki et al., 2004; Sasaki et al., 2009; Babetto et al., 2010). The role of SIRT1 remains controversial, however, as both SIRT1-dependent (Araki et al., 2004) and -independent mechanisms are reported (Wang et al., 2005b).

#### Alzheimer’s disease

Alzheimer’s disease is a terminal neurodegenerative disease, causing neuronal death and brain atrophy. The pathological hallmarks of AD are the intracellular tangles and extracellular plaques in brain. The tangles, also known as neurofibrillary tangles, are formed by accumulation of insoluble tau proteins, and the plaques are deposits of β-amyloid (Aβ) peptides, typically consisting of 40–42 amino acid residues.

The protective effect of SIRT1 against AD was initially observed in CR studies, where CR reduced Aβ and plaque generation in the brains of transgenic AD mice (Patel et al., 2005; Wang et al., 2005a). Similarly, the reduction of Aβ was also noticed in the cortex of fasted squirrel monkeys and is inversely correlated with SIRT1 levels (Qin et al., 2006a). These studies imply that SIRT1 is involved in neuroprotection against AD. Indeed, recent studies demonstrate that SIRT1 activation reduces the neuronal death and brain atrophy that are characteristic of AD (Chen et al., 2005b; Qin et al., 2006b; Kim et al., 2007; Donmez et al., 2010; Min et al., 2010). SIRT1 deficiency is associated with increased levels of phosphorylated-tau in neurons (Min et al., 2010) and the amount of neurofibrillary tangles in AD brains (Julien et al., 2009).

Moreover, recent studies show that either administration of resveratrol or overexpression of SIRT1 reduces Aβ levels both *in vitro* and *in vivo* (Chen et al., 2005b; Qin et al., 2006b; Donmez et al., 2010). SIRT1 overexpression stimulates the production of α-secretase in neurons and mice by two pathways: activating the retinoic acid receptor (RAR; Donmez et al., 2010) and inhibiting the rho-associated, coiled-coil-containing protein kinase 1 (ROCK1; Qin et al., 2006b). Increased levels of α-secretase enhance normal process of Amyloid precursor protein (APP), leading to decreased generation of toxic Aβ. In addition, SIRT1 also reduces the NF-kappaB pathway in microglia and decreases Aβ level (Chen et al., 2005b). Taken together, these results establish that SIRT1 protects against AD by multiple mechanisms, including degradation of tau and reducing levels of Aβ.

#### Parkinson’s disease

Parkinson’s disease is a common neurodegenerative disease caused by the death of dopaminergic neurons of the substantia nigra in the midbrain. The major symptoms of PD are rigidity, tremor, and bradykinesia. Our early study found that CR or use of 2-deoxy-D-glucose, a glucose analog, reduces the loss of dopaminergic neurons in mice and improves motor function, implying that SIRT1 may be involved in the protection (Duan and Mattson, 1999). The levels of SIRT1 in dopaminergic neurons are sharply decreased by treatment with neurotoxins, such as rotenone, 6-hydroxydopamine, α-synuclein, or 1-methyl-4-phenyl-1,2,3,6-tetrahydropyridine (MPTP; Alvira et al., 2007; Pallas et al., 2008; Albani et al., 2009), which are agents widely used to model PD. Additionally, SIRT1 overexpression (Wareski et al., 2009) or activation by resveratrol (Okawara et al., 2007; Chao et al., 2008; Albani et al., 2009) slows neuronal death as well as neurodegeneration in PD models both *in vivo *and *in vitro *(Donmez et al., 2012), indicating a neuroprotective role of SIRT1 against PD. Not all studies showed a protective role of SIRT1, however. For example, no protection was observed in an MPTP-induced PD model in SIRT1 transgenic mice (Kakefuda et al., 2009). Nevertheless, despite the controversy, most research demonstrates a protective role of SIRT1 against PD, although the mechanisms are unclear.

#### Huntington’s disease

Huntington’s disease is an autosomal dominant hereditary disease with onset in middle-age. It is caused by a trinucleotide repeat mutation in the huntingtin gene that results in an increased number of glutamine residues in the N-terminus of the huntingtin protein which causes abnormal protein aggregation and ultimately neuronal death. Our previous study showed that CR could ameliorate the motor phenotype and extend survival of HD mice (Duan et al., 2003), indicating that pathways related to energy metabolism can modify disease progression in the disease. CR increases mitochondrial biogenesis by inducing endothelial nitric oxide synthase (eNOS), and NO can activate the SIRT1 gene (Nisoli et al., 2005; Haigis and Guarente, 2006), which is the mammalian ortholog of yeast Sir2, and a highly conserved NAD^+^-dependent protein deacetylase. Moreover, SIRT1 has been suggested to mediate some beneficial effects of CR (Canto and Auwerx, 2009; Wakeling et al., 2009; Shimokawa and Trindade, 2010; Chalkiadaki and Guarente, 2012).

The first report demonstrating the connection between SIRT1 and HD came from studies by Parker et al. (2005), who found that overexpression of Sir2.1 or treatment with resveratrol rescued neuronal dysfunction phenotypes induced by mutant polyglutamine in *Caenorhabditis elegans*. In contrast to the neuroprotective effect of Sir2.1 in *C. elegans*, Pallos et al. (2008) reported that 50% reduction of Sir2 extended survival and preserved neurons containing photoreceptor in flies expressing mutant Htt. Interestingly, in the fly model system, overexpression of Sir2 does not reduce the lethality or the level of neuronal degeneration caused by mutant Htt. Studies in both *C. elegans* and *Drosophila* suggest that complete loss of Sir2 is deleterious in the worm (Parker et al., 2005) and is deleterious compared with heterozygous loss in mutant Htt-challenged flies (Pallos et al., 2008). Although heterozygous loss of Sir2 is protective in flies, heterozygous loss of Sir2 in worms was not examined. Nevertheless, reduction of Sir2 neither alters the life-span of flies not expressing Htt nor siblings expressing Htt. Several aspects of the role of sirtuins in lifespan in *C. elegans* and *Drosophila *are controversial, and studies have indicated that Sir2 overexpression did not increase lifespan and that dietary restriction increased lifespan in flies independently of dSir2 (Burnett et al., 2011). Nonetheless, overexpression of Sir2 increases the longevity of normal flies and the longevity of diseased flies is slightly increased by elevated Sir2 (Pallos et al., 2008). The different results might be due to the amount of Sir2, its activation status, and different downstream targets involved. These controversial results warrant further investigation of the role of SIRT1 in mammalian systems.

Indeed, two independent studies by our group (Jiang et al., 2011) and Krainc’s group (Jeong et al., 2011) demonstrated that modulating the levels of SIRT1 has therapeutic benefit in three different HD mouse models, and putative downstream targets of SIRT1 involved in improved disease outcomes are also identified. These two studies provide compelling support to the view that SIRT1 provides beneficial effects in HD mouse models, but also raise important questions. It is possible that the contradictory results on the effects of SIRT1 in models of HD might be explained by different effector pathways or mechanisms and by context-dependent effects or different levels of SIRT1 activation. SIRT1 has numerous targets, and different models of HD display different phenotypes by activating various targets and mechanisms. Therefore, it is not surprising to observe contradictory data, especially in different species and different models.

### AMYOTROPHIC LATERAL SCLEROSIS

Amyotrophic lateral sclerosis is a chronic, fatal neurodegenerative disease, characterized pathologically by the death of motor neurons in the spinal cord and cortex, possibly induced by a deficiency in the enzyme superoxide dismutase 1 (SOD1; Rosen, 1993). In the animal model of ALS where a mutant form of SOD1 is expressed, SIRT1 levels are upregulated in motor neurons (Kim et al., 2007). SIRT1 overexpression protects neurons against toxicity induced by the mutant SOD1 in both cultured neurons and mouse brain (Kim et al., 2007). This protection corresponds to the increased deacetylation of p53. Resveratrol also enhances the protective effect of SIRT1 in a mouse model of ALS (Kim et al., 2007; Markert et al., 2010), but multiple doses are appear to be necessary to improve neurological function and increase the longevity of mice (Markert et al., 2010).

#### Multiple sclerosis

Multiple sclerosis is a myelin sheath disease with lesions typically located in the brain, spinal cord or cranial nerves, and, most commonly, in the optic nerve. The causes of multiple sclerosis are not fully identified but likely arise from an autoimmune etiology; therefore, it is traditionally treated as an inflammatory disease. Recently, however, multiple sclerosis has also been considered to be a neurodegenerative disease because of the co-existence of permanent axonal damage, neuronal loss, and neurological disability in patients with the disease (Lassmann, 2010; Shindler et al., 2010). In a mouse model of multiple sclerosis, experimental autoimmune encephalomyelitis (EAE), SIRT1 activation by SRT501 or SRT1720 maintains axonal density, prevents neuronal loss, and improves neuronal dysfunction (Shindler et al., 2007, 2010). SIRT1 inhibition with Sirtinol attenuates the neuroprotective effects of SRT501 (Shindler et al., 2010), suggesting a protective role of SIRT1 in multiple sclerosis. However, further investigations are necessary to fully delineate the role of SIRT1 in multiple sclerosis.

#### Cerebral ischemia

Ischemic stroke is a common neurological disease caused by the sudden reduction or cessation of blood flow to the brain, leading to infarction. The clinical management of stroke is difficult and current drugs must be administered within a limited time window after the onset of the stroke to provide clinical benefit. Promising candidates for neuroprotective strategies include preconditioning, mild hypothermia, and the use of chemical and biological compounds targeting critical molecular mediators of neuronal death and survival.

The neuroprotective effect of SIRT1 was first reported in ischemic preconditioning and the SIRT1 activating compound resveratrol reduced neuronal injury of the hippocampus in global cerebral ischemia in rats. Increased SIRT1 activity was also shown to be a common mechanism for the protective effects of preconditioning and resveratrol (Raval et al., 2006; Morris et al., 2011). Sirtinol, an inhibitor of SIRT1 activity, abolished the neuroprotection of preconditioning and resveratrol (Raval et al., 2006), indicating that SIRT1 plays a key role in mediating neuroprotection. This neuroprotective role is further supported by two recent studies (Chong and Maiese, 2008; Della-Morte et al., 2009) showing that SIRT1 activation reduces ischemic neuronal injuries.

Another study showed that, in primary neuronal culture, pretreatment with NAD^+^ pretreatment markedly reduces neuronal death induced by oxygen-glucose deprivation, an *in vitro* model of ischemia (Wang et al., 2008). SIRT1 is necessary for NAD^+^ neuroprotection, as NAD^+^ treatment upregulates SIRT1 expression and activity, and SIRT1 knockdown attenuates the protection mediated by NAD^+^ (Wang et al., 2008). NAMPT overexpression reduces ischemic infarct, whereas NAMPT inhibition aggravates ischemic injuries. The protective effect of NAMPT is SIRT1-dependent, as SIRT1 knockout blocks the protection (Wang et al., 2011).

Despite the aforementioned evidence, controversy exists over the protective effect of SIRT1 against ischemia. In a study with SIRT1 transgenic mice, where human SIRT1 was overexpressed under the control of rat neuron-specific enolase promoter, no neuroprotection was observed against stroke as SIRT1 and wild-type mice demonstrated almost indistinguishable infarct volumes and neurological deficiency scores (Kakefuda et al., 2009). The discrepancy between this study and the others was probably due to the sustained high level of SIRT1, because it may consume too much or even deplete NAD^+^, which could exaggerate neuronal injury (Wang et al., 2008; Kakefuda et al., 2009; Liu et al., 2009). Therefore, it is possible that NAD^+^ deficiency compromised the neuroprotective effect of SIRT1. In another study, nicotinamide, a compound that inhibits SIRT1 action, showed neuroprotection against ischemic injury, implying that SIRT1 might have a detrimental effect against stroke (Chong et al., 2005). However, this report might overlook other functions of nicotinamide, including that of precursor for NAD^+^ synthesis. In fact, the same group later reported that SIRT1 overexpression prevents neurons from apoptosis after oxidative stress (Chong and Maiese, 2008).

### SIRT1 IN CLINICAL PRACTICE

SIRT1-activating compounds have not yet been proven to be clinically useful for the treatment of neurodegenerative diseases. Preclinical studies have been performed in various neurodegenerative disease models, however. The information obtained from such studies could prove to be valuable for designing SIRT1-activating molecules that may be more likely to be useful clinically. The discovery of such molecules is becoming increasingly important, considering the limitations of genetic manipulations and the lack of unequivocal evidence of specific SIRT1 activation by prototype molecules like resveratrol (Sauve, 2009).

Several rational strategies based on the available protein structure and the catalytic pathways have been designed to develop small molecules that selectively activate sirtuins (Sauve, 2009). One strategy involves designing resveratrol-like molecules, which has not yielded successful results as the *in vivo* mechanism of SIRT1 activation is not fully understood. Another approach aims to increase the cellular levels of NAD+ in order to activate SIRT1 function. This approach has the advantage of harnessing a natural metabolic pathway to enhance SIRT1 functions. Moreover, naturally occurring metabolites present the least risk of toxicity. The efficacies of agents that have been used to enhance NAD^+^ are still questionable, however, and NAD^+^ enhancement affects a host of other physiological pathways, so that the approach is not specific to SIRT1. A third strategy currently in the proof-of-principle stage designated nicotinamide derepression is based on countering the inhibitory effect of nicotinamide on sirtuins by designing molecules that are antagonistic to nicotinamide. This approach is still in its infancy and has not provided compounds with desired potency, but is an attractive strategy to develop further. Details of efforts to discover SIRT1-activating molecules have recently been comprehensively reviewed by Blum et al. (2011).

### FUTURE PERSPECTIVES ON SIRT1

Over the last decade, our understanding of the biology of sirtuins has vastly increased from its original description as an NAD^+^-dependent class III histone deacetylase that can control the lifespan of yeast. Of particular interest is the discovery that SIRT1 not only deacetylates histones, but also some well-known transcriptional regulators, thereby modulating a wide array of biological processes. An exciting aspect is that SIRT1 mediates neuroprotection against both acute and chronic neurological diseases. Importantly, SIRT1 activity is enhanced by small-molecule compounds; therefore, development of small-molecule activators could lead to novel therapies against neurological diseases. One of the broad protective mechanisms of SIRT1 is to suppress genome-wide gene transcription via histone deacetylation. Furthermore, SIRT1 selectively suppresses genes involved in fat storage, apoptosis, and inflammation. Adding to the complexity of SIRT1-mediated cell survival, SIRT1 specifically promotes the transcription of a set of genes related to cell survival, energy metabolism, and mitochondrial biogenesis. SIRT1 thus has multifaceted mechanisms with the end goal to increase cell viability.

Although extensively studied, the biological functions of SIRT1 remain only partially characterized. With respect to mechanism of action, there are several substantial unknowns. For example, it is not known how SIRT1 specifically increases transcription of beneficial genes while it simultaneously suppresses universal transcription. It would be of interest to determine whether similar mechanisms exist for genes upregulated by SIRT1-mediated activation of transcription. Another issue is the paradoxical effect of SIRT1. For example, whereas SIRT1 directly suppresses PPARγ transcriptional activity directly (Picard et al., 2004), it also activates PGC-1α (Nemoto et al., 2005; Rodgers et al., 2005), which could increase the transcriptional activities of PPARγ (Puigserver et al., 1998).

Although SIRT1 has been found to be neuroprotective in numerous studies, it is clear from the diverse pathological mechanisms manifested in neurodegenerative disorders that the role of SIRT1 requires more detailed study. The availability of crystal structures and detailed mechanistic analysis are helpful in discovering SIRT1 modulators, but would be of limited value if they failed to reach clinical trials, thus emphasizing the importance of developing robust animal models for investigating molecular mechanisms involved in SIRT1 activation. Moreover, the potential negative effects of SIRT1 activation and energy depletion need further investigation in animal models. The clinical success of sirtuin activating compounds (STACs) in neurodegenerative diseases relies overwhelmingly on developing new strategies and designing molecules based on the sirtuin chemistry and molecular pathways activated by SIRT1. A very recent study performed by Hubbard et al. (2013) demonstrated that the specific hydrophobic motifs in SIRT1 substrates such as PGC-1α and FOXO3a facilitate SIRT1 activation by STACs, implying that SIRT1 can be directly activated through an allosteric mechanism common to chemically diverse STACs. In summary, there is no doubt that SIRT1 holds promising therapeutic potential for neurodegenerative disorders.

## SIRT3, ENERGY METABOLISM AND AGING

**FIGURE 2:**
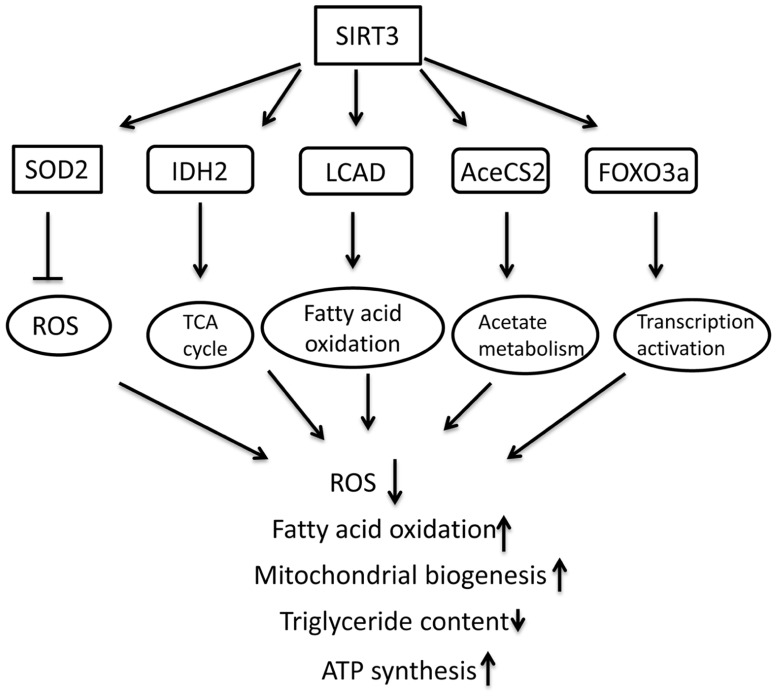
** Schematic overview of molecular targets of SIRT3 and the role in regulating metabolism in mitochondria**.

This section summarizes the studies on the role of mitochondrial SIRT3 in energy metabolism and protection against oxidative stress and age-associated dysfunction (**Figure 2**).

### SIRT3 AND MITOCHONDRIA

Mitochondria are not only the powerhouse for ATP production but also the main sites where reactive oxygen species (ROS) are generated and the intrinsic apoptotic signaling pathway is initiated (Salminen et al., 2008b). The functions of mitochondrial proteins are altered when they are deacetylated by NAD^+^-dependent mitochondrial deacetylases, including SIRT3, SIRT4, and SIRT5. All mitochondrial sirtuins are present in the mitochondrial matrix (Howitz et al., 2003; Rogina and Helfand, 2004). Since mitochondria contain their own DNA, transcription factors, histone-like proteins, and protein synthesis systems, mitochondrial sirtuins deacetylate a set of targets within the mitochondria that are distinct from those of nuclear proteins (Tissenbaum and Guarente, 2001; Wood et al., 2004). Although precise mechanistic information is still lacking, evidence is emerging to suggest that mitochondrial sirtuins protect against oxidative stress (Braunstein et al., 1993; Baur et al., 2006).

Among the mitochondrial sirtuins, SIRT3 functions have been characterized in the greatest detail. Initial studies of SIRT3-deficient mice indicated that loss of SIRT3, but not SIRT4 or SIRT5, led to dramatic protein hyperacetylation within mitochondria, suggesting that SIRT3 is the major mitochondrial deacetylase activity (Dali-Youcef et al., 2007). In humans, full-length SIRT3 is a 44-kD protein with an N-terminal mitochondrial targeting sequence that is an enzymatically inactive *in vitro*. It is proteolytically processed in mitochondria to a mature 28 kD catalytically active deacetylase (Onyango et al., 2002; Schwer et al., 2002). The first mouse SIRT3 cDNA sequence identified encoded a 28-kD protein lacking the N-terminal mitochondrial targeting sequence (Yang et al., 2000). Several recent studies have identified a longer isoform of murine SIRT3 encoding a 37 kD protein, however, that can be imported into mitochondria and processed into the mature 28 kD protein (Cooper et al., 2009; Jin et al., 2009; Bao et al., 2010; Yang et al., 2010). Whether or not an active fraction of SIRT3 exists outside mitochondria and modifies extra-mitochondrial proteins remains controversial.

### SIRT3 AND METABOLIC HOMEOSTASIS

Emerging data have shown that one major function of SIRT3 is regulation of mitochondrial electron transport chain activity to maintain energy homeostasis. The main energy source in mitochondria is pyruvate, a product of glycolysis. Alternatively, mitochondria also burn fatty acids, amino acids, and acetates when pyruvate is deficient. For fatty acid catabolism, long-chain acyl coenzyme A dehydrogenase (LCAD) is a key enzyme that breaks down fatty acids and generates acetyl-CoA, stimulating β-oxidation. In SIRT3 knockout mice, LCAD is hyperacetylated at Lys42, leading to decreases in enzymatic activity, β-oxidation, and ATP level (Hirschey et al., 2010). Interestingly, these mice do not tolerate cold exposure during fasting (Hirschey et al., 2010). SIRT3 directly deacetylates LACD at Lys42 and increases LACD activity (Hirschey et al., 2010). In addition, SIRT3 may promote β-oxidation via multiple mechanisms, such as by deacetylating other β-oxidation enzymes, including the short-chain L-3-hydroxyacyl-CoA dehydrogenase and the very-long-chain acyl coenzyme A dehydrogenase (Hallows et al., 2011), facilitating mitochondrial adaptation to fuel changes.

Glucose is the major energy source for cells. When its availability is limited, however, alternative fuels become increasingly important for cell survival. The first step of glycolysis is the conversion of glucose to glucose-6-phosphate, a reaction catalyzed by hexokinases. It is reported that SIRT3 deacetylates cyclophilin D (Hafner et al., 2010; Shulga et al., 2010), which leads to the dissociation of hexokinase II and mitochondria, decreases glucose metabolism, and stimulates oxidative phosphorylation (Shulga et al., 2010).

Acetate derived from acetic acid and alcohol is also used as a mitochondrial fuel, although this only occurs in extreme circumstances of nutrient depletion. In the initial step acetate is converted to acetyl-CoA catalyzed by acetyl-CoA synthetases. Acetyl-CoA synthetase 2 is the mitochondrial form of the enzyme. SIRT3 deacetylates acetyl-CoA synthetase 2 and enhances its activity, leading to increased production of acetyl-CoA (Hallows et al., 2006; Schwer et al., 2006). The acetyl-CoA from fatty acids and acetates as well as α-ketoglutarate from amino acids can enter the Krebs cycle. These two reactions are enhanced by SIRT3 (Hallows et al., 2006; Schwer et al., 2006; Lombard et al., 2007; Schlicker et al., 2008). Additionally, SIRT3 directly stimulates the Krebs cycle. The third step of the cycle is the conversion of 6-carbon isocitrate to 5-carbon α-ketoglutarate, a process catalyzed by isocitrate dehydrogenase 2 (IDH2). A recent study shows that SIRT3 directly deacetylates this dehydrogenase to increase its activity (Someya et al., 2010).

NADH dehydrogenase 1 alpha subcomplex subunit 9 (NDUFA9) is an enzyme of mitochondrial complex I that is acetylated at Lys370 (Kim et al., 2006). SIRT3 physically interacts with NDUFA9 and deacetylates it. SIRT3 knockout enhances its acetylation and reduces the activity of complex I (Ahn et al., 2008), indicating that SIRT3 is a positive regulator of complex I. Complex II, also known as succinate dehydrogenase, is composed of four subunits, including the flavoprotein succinate dehydrogenase subunit A (SdhA). In SIRT3 knockout mice, SdhA is hyperacetylated at several lysine residues, and shows decreased activity of complex II (Cimen et al., 2010). SIRT3 overexpression reverses the acetylation of SdhA and increases complex II activity (Cimen et al., 2010), indicating that SdhA is a SIRT3 substrate, and that SIRT3 is also a positive regulator of complex II.

SIRT3 is also reported to bind the alpha subunit 1 of the F1 particle of ATP synthase (Law et al., 2009), but the function is unclear. Taken together, these results suggest that SIRT3 promotes ATP generation through enhancing action of several enzymes involved in energy metabolism. Further supporting this role, SIRT3-knockout mice show substantial acetylation of mitochondrial proteins, and have reduced ATP levels at baseline and during cellular stress (Ahn et al., 2008).

Mitochondria are also the major sites for the generation of the ROS, superoxide, and also where the superoxide is dismuted by mitochondrial MnSOD. Recent reports show that SIRT3 deacetylates MnSOD at Lys122 and increases its activity, reducing oxidative and radiation stress in mice (Qiu et al., 2010; Tao et al., 2010). Overexpression of SIRT3 protects HEK293 from oxidative stress and prevents age-related cochlear cell death in mice (Someya et al., 2010). Overall, this suggests anti-oxidative and neuroprotective roles of SIRT3. New data suggest that the SIRT3 deacetylase plays a key role in bolstering mitochondrial anti-oxidant defenses during CR (Qiu et al., 2010; Someya et al., 2010).

In future studies, it will be of interest to define the mechanism whereby acetylation of electron transport chain subunits affects generation of ATP. It is also important to elucidate why it might be desirable under some physiologic conditions to downregulate electron transport chain activity by increased acetylation. As an added wrinkle, SIRT3 negatively regulates translation within mitochondria by deacetylating the ribosomal protein MRPL10, a function proposed to reduce respiration (Yang et al., 2010).

SIRT3 levels are increased in adipose tissue, skeletal muscle, and liver during CR (Shi et al., 2005; Palacios et al., 2009; Schwer et al., 2009; Hirschey et al., 2010), and conversely decline in response to high-fat feeding (Palacios et al., 2009; Bao et al., 2010; Kendrick et al., 2011). These expression data suggest that SIRT3 might play a role in the response to metabolic homeostasis. In mammals, two independent studies showed that SIRT3 interacts with and deacetylates acetyl-CoA synthetase 2 (AceCS2) at the active site lysine to promote AceCS2 activity (Hallows et al., 2006; Schwer et al., 2006). Under fed conditions, the majority of acetyl-CoA is generated through metabolism of pyruvate by Pyruvate dehydrogenase complex (PDC) and by fatty acid β-oxidation, largely bypassing the need for AceCS2. In this regard, studies of AceCS2-deficient mice revealed that AceCS2 is specifically required for metabolic homeostasis when the mice are fed a low carbohydrate/high fat diet (LC/HFD); AceCS2-deficient animals are essentially normal on a chow diet but show poor weight gain, hypothermia, hypoglycemia, and impaired survival on an LC/HFD (Sakakibara et al., 2009). Presumably the role of SIRT3 in regulating AceCS2 could also be important during fasting, when acetate can be used as a source of energy in extrahepatic tissues (Hirschey et al., 2010). Indeed, SIRT3 has recently been shown to deacetylate and activate 3-hydroxy-3-methylglutaryl-CoA synthase 2 (HMGCS2), a mitochondrial enzyme that converts acetyl-CoA into ketone bodies (acetoacetate, β-hydroxybutyrate, and acetone) in the liver under fasting conditions, which can in turn be used as a source of energy in certain tissues including the brain (Shimazu et al., 2010). SIRT3-deficient mice are unable to produce normal levels of ketone bodies upon fasting.

### SIRT, LIFESPAN AND AGE-ASSOCIATED PHENOTYPES

The role of SIRT3 in aging is of considerable interest because it appears to suppress ROS, one of the causes contributing to the process of aging. In addition to elucidating its roles in regulating specific biochemical pathways in mitochondria, there is great interest in testing whether SIRT3 might modulate age-associated phenotypes, or indeed lifespan itself. In this regard, some studies have linked polymorphisms in the SIRT3 genomic locus to human longevity, although others have failed to demonstrate this association (Rose et al., 2003; Bellizzi et al., 2005, 2007; Lescai et al., 2009). A polymorphism associated with decreased SIRT3 mRNA expression was present in cohorts of young but not old men, suggesting that reduced SIRT3 expression may be detrimental to survival in old age (Bellizzi et al., 2005, 2009). In sedentary individuals, SIRT3 protein expression declined with age in skeletal muscle mitochondria, concomitant with a reduction in respiratory function (Lanza et al., 2008).

Age-related hearing loss (ARHL) is a common problem in the elderly, occurring secondary to cell loss and other degenerative changes in the cochlea. An elegant study has firmly established a role for SIRT3 in preventing ARHL (Someya et al., 2010). One mechanism by which SIRT3 mediates this effect is by deacetylation of IDH2 (Schlicker et al., 2008; Someya et al., 2010), which converts isocitrate to α-ketoglutarate concomitant with reduction of NADP^+^. NADPH in turn allows regeneration of reduced glutathione to promote mitochondrial oxidative defense. In response to CR, wild-type mice, but not SIRT3-deficient animals, show increased NADPH levels, increased reduced glutathione in mitochondria, and decreased DNA damage in the cochlea and in other tissues. In tissue culture cells, overexpression of SIRT3 or IDH2 protects against oxidative stress-induced cell death, and the two proteins together have a synergistic pro-survival effect. These results do not rule out the possibility that SIRT3 might modify other substrates in addition to IDH2 to prevent AHRL during CR. Similarly, Qiu et al. (2010) reported that SIRT3-deficient mice fail to suppress ROS levels and macromolecular damage during CR. They find that SIRT3 directly deacetylates SOD2 to increase its activity during CR, whereas SIRT3-deficient mice do not show SOD2 deacetylation in response to this diet (Qiu et al., 2010). Overall, these papers point to crucial role for SIRT3 in suppressing oxidative damage and its negative sequel during CR. It remains to be seen how SIRT3, or the other mitochondrial sirtuins, might affect other phenotypes of aging and/or effects of CR. The reduction of serum insulin and triglycerides that normally occurs during CR is not observed in SIRT3-deficient mice (Someya et al., 2010), implying that SIRT3 plays additional, uncharacterized roles in the adaptation to this dietary regimen. A recent study indicates that upregulation of SIRT3 indeed reverses aging-associated degeneration in hematopoietic stem cells (Brown et al., 2013), and SIRT3 may promote organismal longevity by maintaining the integrity of tissue-specific stem cells.

### SIRT3 AND MITOCHONDRIAL PROTEIN ACETYLATION: UNRESOLVED QUESTIONS

Acetylation of mitochondrial proteins plays a major role in regulating functions of this organelle. Despite the rapid progress in this area, there are still many outstanding questions remain that will no doubt provide fruitful avenues for research for years to come. In particular, how mitochondrial proteins are acetylated in the first place is currently unknown. The identity of putative mitochondrial acetyltransferases remains elusive; identification of such proteins would represent a major step forward in this field. Alternatively, or in addition to enzymatic acetylation within mitochondria, mitochondrial proteins could in principle be acetylated outside this organelle, before or concomitant with mitochondrial import or even be acetylated non-enzymatically. These latter models would not permit rapid cycles of acetylation/deacetylation of mitochondrial proteins to regulate target protein function in response to varied environmental challenges. Instead, after deacetylation, restoration of acetylation status would require new protein synthesis. Such models could be distinguished through pulse-chase experiments assessing acetylation of newly synthesized mitochondrial proteins before and after mitochondrial import.

Similarly, how SIRT3 activity is regulated in the mitochondria is incompletely understood. SIRT3 requires NAD^+^, and therefore mitochondrial NAD^+^ levels play a critically important role in governing mitochondrial SIRT3 function. Increased NADH generation from NAD^+^ that occurs with HFD and leads to reduced SIRT3 function may explain the increased global mitochondrial protein acetylation observed during this diet, as could increase levels of acetyl-CoA, the substrate for acetyltransferases (Kendrick et al., 2011). This overall increased acetylation may represent the net effect of increased acetyltransferase activity superimposed upon elevated SIRT3 function; alternatively, some protein species hyperacetylated during CR or other conditions may not be substrates for mitochondrial SIRT3. The activity of SIRT3 and other mitochondrial sirtuins might be influenced by other conditions in addition to NAD^+^ levels, such as post-translational modification or interactions with regulatory proteins.

It is currently unclear whether interventions that have an impact on acetylation of many mitochondrial proteins – SIRT3 deficiency, such as CR, HFD – lead to modification of common sets of proteins on the same lysine sites, or whether this response is tailored to different environmental perturbations. Similarly, it remains unclear whether the mitochondrial sirtuins share common targets and/or functions in common. This question could be addressed in mice or cells with mitochondrial sirtuin deficiencies or knockdowns. Given that SIRT3 deacetylates many proteins in mitochondria as well as suppresses some age-associated phenotypes, it will be of interest to test whether acetylation of mitochondrial proteins changes with age, either individually or globally, and whether prevention of this effect might have a beneficial effect on health span or even lifespan.

In addition, whereas the functional impact of acetylation on a few individual protein targets is clear, a global understanding of how SIRT3 affects the activity of metabolic pathways in mitochondria, cells, tissues, and the organism overall is still lacking. Answers to these and related questions involving SIRT3 and mitochondrial protein acetylation will no doubt reveal novel aspects of mitochondrial biology, and perhaps ultimately provide the basis for novel therapeutic strategies for a variety of disorders.

## FURTHER PERSPECTIVES

In the past decade, the function of mammalian sirtuins has been investigated in greater detail than ever before, and we now have a much better molecular understanding of the multiple roles this unique family of enzymes plays in seemingly every biological process. There is little doubt that sirtuins have emerged as critical modulators of metabolic adaptive responses, and their activities have been linked to metabolic abnormalities as well as age-associated neurodegeneration. Yet, key questions will keep investigators busy in the coming years. We still have poor understanding of the molecular mechanisms regulating expression and activity of the sirtuins, and of the precise stimuli that regulate these proteins, and whether the activities of different sirtuins are regulated in a coordinated fashion. In other words, is there cross-talk between sirtuins? Will future studies cement the argument that sirtuins are, indeed, critical modulators of lifespan? This review has focused on SIRT1 and SIRT3. Even less is known about other sirtuins. Further investigation into the targets and functions of this unique family of sirtuins will help develop new strategies for protection against and recovery from common aging-related neurological diseases and promote successful aging.

## Conflict of Interest Statement

The author declares that the research was conducted in the absence of any commercial or financial relationships that could be construed as a potential conflict of interest.

## References

[B1] Adler A. S. Sinha S. Kawahara T. L. Zhang J. Y. Segal E. Chang H. Y. (2007). Motif module map reveals enforcement of aging by continual NF-kappaB activity. *Genes Dev.* 21 3244–3257 10.1101/gad.1588507 18055696 PMC2113026

[B2] Ahn B. H. Kim H. S. Song S. Lee I. H. Liu J. Vassilopoulos A. (2008). A role for the mitochondrial deacetylase Sirt3 in regulating energy homeostasis. *Proc. Natl. Acad. Sci. U.S.A.* 105 14447–14452 10.1073/pnas.0803790105 18794531 PMC2567183

[B3] Albani D. Polito L. Batelli S. De Mauro S. Fracasso C. Martelli G. (2009). The SIRT1 activator resveratrol protects SK-N-BE cells from oxidative stress and against toxicity caused by alpha-synuclein or amyloid-beta (1–42) peptide. *J. Neurochem.* 110 1445–1456 10.1111/j.1471-4159.2009.06228.x 19558452

[B4] Alvira D. Yeste-Velasco M. Folch J. Verdaguer E. Canudas A. M. Pallas M. (2007). Comparative analysis of the effects of resveratrol in two apoptotic models: inhibition of complex I and potassium deprivation in cerebellar neurons. *Neuroscience* 147 746–756 10.1016/j.neuroscience.2007.04.029 17583434

[B5] Araki T. Sasaki Y. Milbrandt J. (2004). Increased nuclear NAD biosynthesis and SIRT1 activation prevent axonal degeneration. *Science* 305 1010–1013 10.1126/science.1098014 15310905

[B6] Babetto E. Beirowski B. Janeckova L. Brown R. Gilley J. Thomson D. (2010). Targeting NMNAT1 to axons and synapses transforms its neuroprotective potency in vivo. *J. Neurosci.* 30 13291–13304 10.1523/JNEUROSCI.1189-10.2010 20926655 PMC6634738

[B7] Bao J. Scott I. Lu Z. Pang L. Dimond C. C. Gius D. (2010). SIRT3 is regulated by nutrient excess and modulates hepatic susceptibility to lipotoxicity. *Free Radic. Biol. Med.* 49 1230–1237 10.1016/j.freeradbiomed.2010.07.009 20647045 PMC2943385

[B8] Baur J. A. Pearson K. J. Price N. L. Jamieson H. A. Lerin C. Kalra A. (2006). Resveratrol improves health and survival of mice on a high-calorie diet. *Nature* 444 337–342 10.1038/nature05354 17086191 PMC4990206

[B9] Bellizzi D. Covello G. Di Cianni F. Tong Q De Benedictis G. (2009). Identification of GATA2 and AP-1 activator elements within the enhancer VNTR occurring in intron 5 of the human SIRT3 gene. *Mol. Cells* 28 87–92 10.1007/s10059-009-0110-3 19714312

[B10] Bellizzi D. Dato S. Cavalcante P. Covello G. Di Cianni F. Passarino G. (2007). Characterization of a bidirectional promoter shared between two human genes related to aging: SIRT3 and PSMD13. *Genomics* 89 143–150 10.1016/j.ygeno.2006.09.004 17059877

[B11] Bellizzi D. Rose G. Cavalcante P. Covello G. Dato S. De Rango F. (2005). A novel VNTR enhancer within the SIRT3 gene, a human homologue of SIR2, is associated with survival at oldest ages. *Genomics* 85 258–263 10.1016/j.ygeno.2004.11.003 15676284

[B12] Biessels G. J. Kappelle L. J. (2005). Increased risk of Alzheimer’s disease in Type II diabetes: insulin resistance of the brain or insulin-induced amyloid pathology? *Biochem. Soc. Trans.* 33 1041–1044 10.1042/BST20051041 16246041

[B13] Blum C. A. Ellis J. L. Loh C. Ng P. Y. Perni R. B. Stein R. L. (2011). SIRT1 modulation as a novel approach to the treatment of diseases of aging. *J. Med. Chem.* 54 417–432 10.1021/jm100861p 21080630

[B14] Boveris A. Navarro A. (2008). Brain mitochondrial dysfunction in aging. *IUBMB Life* 60 308–314 10.1002/iub.46 18421773

[B15] Braunstein M. Rose A. B. Holmes S. G. Allis C. D. Broach J. R. (1993). Transcriptional silencing in yeast is associated with reduced nucleosome acetylation. *Genes Dev.* 7 592–604 10.1101/gad.7.4.592 8458576

[B16] Brooks C. L. Gu W. (2009). How does SIRT1 affect metabolism, senescence and cancer? *Nat. Rev. Cancer* 9 123–128 10.1038/nrc2562 19132007 PMC2857763

[B17] Brown K. Xie S. Qiu X. Mohrin M. Shin J. Liu Y. (2013). SIRT3 reverses aging-associated degeneration. *Cell Rep.* 3 319–327 10.1016/j.celrep.2013.01.005 23375372 PMC3582834

[B18] Burnett C. Valentini S. Cabreiro F. Goss M. Somogyvari M. Piper M. D. (2011). Absence of effects of Sir2 overexpression on lifespan in* C. elegans* and *Drosophila*. *Nature* 477 482–485 10.1038/nature10296 21938067 PMC3188402

[B19] Campisi J. (2005). Suppressing cancer: the importance of being senescent. *Science* 309 886–887 10.1126/science.1116801 16081723

[B20] Canto C. Auwerx J. (2009). Caloric restriction, SIRT1 and longevity. *Trends Endocrinol. Metab.* 20 325–331 10.1016/j.tem.2009.03.008 19713122 PMC3627124

[B21] Chalkiadaki A. Guarente L. (2012). Sirtuins mediate mammalian metabolic responses to nutrient availability. *Nat. Rev. Endocrinol.* 8 287–296 10.1038/nrendo.2011.225 22249520

[B22] Chao J. Yu M. S. Ho Y. S. Wang M. Chang R. C. (2008). Dietary oxyresveratrol prevents parkinsonian mimetic 6-hydroxydopamine neurotoxicity. *Free Radic. Biol. Med.* 45 1019–1026 10.1016/j.freeradbiomed.2008.07.002 18675900

[B23] Chen D. Steele A. D. Lindquist S. Guarente L. (2005a). Increase in activity during calorie restriction requires Sirt1. *Science* 310 1641 10.1126/science.1118357 16339438

[B24] Chen J. Zhou Y. Mueller-Steiner S. Chen L. F. Kwon H. Yi S. (2005b). SIRT1 protects against microglia-dependent amyloid-beta toxicity through inhibiting NF-kappaB signaling. *J. Biol. Chem.* 280 40364–40374 10.1074/jbc.M509329200 16183991

[B25] Chen L. F. Greene W. C. (2003). Regulation of distinct biological activities of the NF-kappaB transcription factor complex by acetylation. *J. Mol. Med. (Berl.)* 81 549–57 10.1007/s00109-003-0469-0 12920522

[B26] Chong Z. Z. Lin S. H. Li F. Maiese K. (2005). The sirtuin inhibitor nicotinamide enhances neuronal cell survival during acute anoxic injury through AKT, BAD, PARP, and mitochondrial associated “anti-apoptotic” pathways. *Curr. Neurovasc. Res.* 2 271–285 10.2174/156720205774322584 16181120 PMC1986682

[B27] Chong Z. Z. Maiese K. (2008). Enhanced tolerance against early and late apoptotic oxidative stress in mammalian neurons through nicotinamidase and sirtuin mediated pathways. *Curr. Neurovasc. Res.* 5 159–170 10.2174/156720208785425666 18691073 PMC2615543

[B28] Cimen H. Han M. J. Yang Y. Tong Q. Koc H. Koc E. C. (2010). Regulation of succinate dehydrogenase activity by SIRT3 in mammalian mitochondria. *Biochemistry* 49 304–311 10.1021/bi901627u 20000467 PMC2826167

[B29] Cooper H. M. Huang J. Y. Verdin E. Spelbrink J. N. (2009). A new splice variant of the mouse SIRT3 gene encodes the mitochondrial precursor protein. *PLoS ONE* 4:e4986 10.1371/journal.pone.0004986 PMC2659428 19333382

[B30] Dali-Youcef N. Lagouge M. Froelich S. Koehl C. Schoonjans K. Auwerx J. (2007). Sirtuins: the ‘magnificent seven’, function, metabolism and longevity. *Ann. Med.* 39 335–345 10.1080/07853890701408194 17701476

[B31] Della-Morte D. Dave K. R. DeFazio R. A. Bao Y. C. Raval A. P. Perez-Pinzon M. A. (2009). Resveratrol pretreatment protects rat brain from cerebral ischemic damage via a sirtuin 1-uncoupling protein 2 pathway. *Neuroscience* 159 993–1002 10.1016/j.neuroscience.2009.01.017 19356683 PMC2668125

[B32] Dietrich M. O. Antunes C. Geliang G. Liu Z. W. Borok E. Nie Y. (2010). Agrp neurons mediate Sirt1’s action on the melanocortin system and energy balance: roles for Sirt1 in neuronal firing and synaptic plasticity. *J. Neurosci.* 30 11815–11825 10.1523/JNEUROSCI.2234-10.2010 20810901 PMC2965459

[B33] Donmez G. Arun A. Chung C. Y. McLean P. J. Lindquist S. Guarente L. (2012). SIRT1 protects against alpha-synuclein aggregation by activating molecular chaperones. *J. Neurosci.* 32 124–132 10.1523/JNEUROSCI.3442-11.2012 22219275 PMC3263206

[B34] Donmez G. Wang D. Cohen D. E. Guarente L. (2010). SIRT1 suppresses beta-amyloid production by activating the alpha-secretase gene ADAM10. *Cell* 142 320–332 10.1016/j.cell.2010.06.020 20655472 PMC2911635

[B35] Drew B. Leeuwenburgh C. (2004). Ageing and subcellular distribution of mitochondria: role of mitochondrial DNA deletions and energy production. *Acta Physiol. Scand.* 182 333–341 10.1111/j.1365-201X.2004.01371.x 15569094

[B36] Duan W. Guo Z. Jiang H. Ware M. Li X. J. Mattson M. P. (2003). Dietary restriction normalizes glucose metabolism and BDNF levels, slows disease progression, and increases survival in huntingtin mutant mice. *Proc. Natl. Acad. Sci. U.S.A.* 100 2911–2916 10.1073/pnas.0536856100 12589027 PMC151440

[B37] Duan W. Mattson M. P. (1999). Dietary restriction and 2-deoxyglucose administration improve behavioral outcome and reduce degeneration of dopaminergic neurons in models of Parkinson’s disease. *J. Neurosci. Res.* 57 195–206 10.1002/(SICI)1097-4547(19990715)57:2 10398297

[B38] Finley L. W. Haigis M. C. (2009). The coordination of nuclear and mitochondrial communication during aging and calorie restriction. *Ageing Res. Rev.* 8 173–188 10.1016/j.arr.2009.03.003 19491041 PMC4702504

[B39] Guarente L. Franklin H. (2011). Epstein lecture: sirtuins, aging, and medicine. *N. Engl. J. Med.* 364 2235–2244 10.1056/NEJMra1100831 21651395

[B40] Hafner A. V. Dai J. Gomes A. P. Xiao C. Y. Palmeira C. M. Rosenzweig A. (2010). Regulation of the mPTP by SIRT3-mediated deacetylation of CypD at lysine 166 suppresses age-related cardiac hypertrophy. *Aging (Albany NY)* 2 914–923 21212461 10.18632/aging.100252 PMC3034180

[B41] Haigis M. C. Guarente L. P. (2006). Mammalian sirtuins – emerging roles in physiology, aging, and calorie restriction. *Genes Dev.* 20 2913–2921 10.1101/gad.1467506 17079682

[B42] Hallows W. C. Lee S. Denu J. M. (2006). Sirtuins deacetylate and activate mammalian acetyl-CoA synthetases. *Proc. Natl. Acad. Sci. U.S.A.* 103 10230–10235 10.1073/pnas.0604392103 16790548 PMC1480596

[B43] Hallows W. C. Yu W. Smith B. C. Devries M. K. Ellinger J. J. Someya S. (2011). Sirt3 promotes the urea cycle and fatty acid oxidation during dietary restriction. *Mol. Cell.* 41 139–149 10.1016/j.molcel.2011.01.002 21255725 PMC3101115

[B44] Hardie D. G. (2011). AMP-activated protein kinase: an energy sensor that regulates all aspects of cell function. *Genes Dev.* 25 1895–1908 10.1101/gad.17420111 21937710 PMC3185962

[B45] Herranz D. Munoz-Martin M. Canamero M. Mulero F. Martinez-Pastor B. Fernandez-Capetillo O. (2010). Sirt1 improves healthy ageing and protects from metabolic syndrome-associated cancer. *Nat. Commun.* 1 3 10.1038/ncomms1001 PMC3641391 20975665

[B46] Hirschey M. D. Shimazu T. Goetzman E. Jing E. Schwer B. Lombard D. B. (2010). SIRT3 regulates mitochondrial fatty-acid oxidation by reversible enzyme deacetylation. *Nature* 464 121–125 10.1038/nature08778 20203611 PMC2841477

[B47] Howitz K. T. Bitterman K. J. Cohen H. Y. Lamming D. W. Lavu S. Wood J. G. (2003). Small molecule activators of sirtuins extend *Saccharomyces cerevisiae* lifespan. *Nature* 425 191–196 10.1038/nature01960 12939617

[B48] Hubbard B. P. Gomes A. P. Dai H. Li J. Case A. W. Considine T. (2013). Evidence for a common mechanism of SIRT1 regulation by allosteric activators. *Science* 339 1216–1219 10.1126/science.1231097 23471411 PMC3799917

[B49] Imai S. Guarente L. (2010). Ten years of NAD-dependent SIR2 family deacetylases: implications for metabolic diseases. *Trends Pharmacol. Sci.* 31 212–220 10.1016/j.tips.2010.02.003 20226541 PMC3526941

[B50] Jeong H. Cohen D. E. Cui L. Supinski A. Savas J. N. Mazzulli J. R. (2011). Sirt1 mediates neuroprotection from mutant huntingtin by activation of the TORC1 and CREB transcriptional pathway. *Nat. Med.* 18 159–165 10.1038/nm.2559 22179316 PMC3509213

[B51] Jiang J. C. Jaruga E. Repnevskaya M. V. Jazwinski S. M. (2000). An intervention resembling caloric restriction prolongs life span and retards aging in yeast. *FASEB J.* 14 2135–2137 10.1096/fj.00-0242fje 11024000

[B52] Jiang M. Wang J. Fu J. Du L. Jeong H. West T. (2011). Neuroprotective role of Sirt1 in mammalian models of Huntington’s disease through activation of multiple Sirt1 targets. *Nat. Med.* 18 153–158 10.1038/nm.2558 22179319 PMC4551453

[B53] Jin L. Galonek H. Israelian K. Choy W. Morrison M. Xia Y. (2009). Biochemical characterization, localization, and tissue distribution of the longer form of mouse SIRT3. *Protein Sci.* 18 514–525 10.1002/pro.50 19241369 PMC2760358

[B54] Julien C. Tremblay C. Emond V. Lebbadi M. Salem N. Jr. Bennett D. A. (2009). Sirtuin 1 reduction parallels the accumulation of tau in Alzheimer disease. *J. Neuropathol. Exp. Neurol.* 68 48–58 10.1097/NEN.0b013e3181922348 19104446 PMC2813570

[B55] Jung C. H. Ro S. H. Cao J. Otto N. M. Kim D. H. (2010). mTOR regulation of autophagy. *FEBS Lett.* 584 1287–1295 10.1016/j.febslet.2010.01.017 20083114 PMC2846630

[B56] Kaeberlein M. McVey M. Guarente L. (1999). The SIR2/3/4 complex and SIR2 alone promote longevity in *Saccharomyces cerevisiae* by two different mechanisms. *Genes Dev.* 13 2570–2580 10.1101/gad.13.19.2570 10521401 PMC317077

[B57] Kakefuda K. Fujita Y. Oyagi A. Hyakkoku K. Kojima T. Umemura K. (2009). Sirtuin 1 overexpression mice show a reference memory deficit, but not neuroprotection. *Biochem. Biophys. Res. Commun.* 387 784–788 10.1016/j.bbrc.2009.07.119 19643082

[B58] Kendrick A. A. Choudhury M. Rahman S. M. McCurdy C. E. Friederich M. Van Hove J. L. (2011). Fatty liver is associated with reduced SIRT3 activity and mitochondrial protein hyperacetylation. *Biochem. J.* 433 505–514 10.1042/BJ20100791 21044047 PMC3398511

[B59] Kim D. Nguyen M. D. Dobbin M. M. Fischer A. Sananbenesi F. Rodgers J. T. (2007). SIRT1 deacetylase protects against neurodegeneration in models for Alzheimer’s disease and amyotrophic lateral sclerosis. *EMBO J.* 26 3169–3179 10.1038/sj.emboj.7601758 17581637 PMC1914106

[B60] Kim S. C. Sprung R. Chen Y. Xu Y. Ball H. Pei J. (2006). Substrate and functional diversity of lysine acetylation revealed by a proteomics survey. *Mol. Cell.* 23 607–618 10.1016/j.molcel.2006.06.026 16916647

[B61] Klar A. J. Fogel S. (1979). Activation of mating type genes by transposition in *Saccharomyces cerevisiae*. *Proc. Natl. Acad. Sci. U.S.A.* 76 4539–4543 10.1073/pnas.76.9.4539 388445 PMC411613

[B62] Koubova J. Guarente L. (2003). How does calorie restriction work? *Genes Dev.* 17 313–321 10.1101/gad.1052903 12569120

[B63] Lan F. Cacicedo J. M. Ruderman N. Ido Y. (2008). SIRT1 modulation of the acetylation status, cytosolic localization, and activity of LKB1. Possible role in AMP-activated protein kinase activation. *J. Biol. Chem.* 283 27628–27635 10.1074/jbc.M805711200 18687677 PMC2562073

[B64] Lanza I. R. Short D. K. Short K. R. Raghavakaimal S. Basu R. Joyner M. J. (2008). Endurance exercise as a countermeasure for aging. *Diabetes* 57 2933–2942 10.2337/db08-0349 18716044 PMC2570389

[B65] Lassmann H. (2010). Axonal and neuronal pathology in multiple sclerosis: what have we learnt from animal models. *Exp. Neurol.* 225 2–8 10.1016/j.expneurol.2009.10.009 19840788

[B66] Lavu S. Boss O. Elliott P. J. Lambert P. D. (2008). Sirtuins – novel therapeutic targets to treat age-associated diseases. *Nat. Rev. Drug Discov.* 7 841–853 10.1038/nrd2665 18827827

[B67] Law I. K. Liu L. Xu A. Lam K. S. Vanhoutte P. M. Che C. M. (2009). Identification and characterization of proteins interacting with SIRT1 and SIRT3: implications in the anti-aging and metabolic effects of sirtuins. *Proteomics* 9 2444–2456 10.1002/pmic.200800738 19343720

[B68] Lee I. H. Cao L. Mostoslavsky R. Lombard D. B. Liu J. Bruns N. E. (2008). A role for the NAD-dependent deacetylase Sirt1 in the regulation of autophagy. *Proc. Natl. Acad. Sci. U.S.A.* 105 3374–3379 10.1073/pnas.0712145105 18296641 PMC2265142

[B69] Lescai F. Blanche H. Nebel A. Beekman M. Sahbatou M. Flachsbart F. (2009). Human longevity and 11p15.5: a study in 1321 centenarians. *Eur. J. Hum. Genet.* 17 1515–1519 10.1038/ejhg.2009.54 19367319 PMC2986679

[B70] Liu D. Gharavi R. Pitta M. Gleichmann M. Mattson M. P. (2009). Nicotinamide prevents NAD^+^ depletion and protects neurons against excitotoxicity and cerebral ischemia: NAD^+^ consumption by SIRT1 may endanger energetically compromised neurons. *Neuromol. Med.* 11 28–42 10.1007/s12017-009-8058-1 PMC2677622 19288225

[B71] Lombard D. B. Alt F. W. Cheng H. L. Bunkenborg J. Streeper R. S. Mostoslavsky R. (2007). Mammalian Sir2 homolog SIRT3 regulates global mitochondrial lysine acetylation. *Mol. Cell. Biol.* 27 8807–8814 10.1128/MCB.01636-07 17923681 PMC2169418

[B72] Luo J. Nikolaev A. Y. Imai S. Chen D. Su F. Shiloh A. (2001). Negative control of p53 by Sir2alpha promotes cell survival under stress. *Cell* 107 137–148 10.1016/S0092-8674(01)00524-4 11672522

[B73] Madeo F. Tavernarakis N. Kroemer G. (2010). Can autophagy promote longevity? *Nat. Cell Biol.* 12 842–846 10.1038/ncb0910-842 20811357

[B74] Markert C. D. Kim E. Gifondorwa D. J. Childers M. K. Milligan C. E. (2010). A single-dose resveratrol treatment in a mouse model of amyotrophic lateral sclerosis. *J. Med. Food* 13 1081–1085 10.1089/jmf.2009.0243 20626250

[B75] Masoro E. J. (2000). Caloric restriction and aging: an update. *Exp. Gerontol.* 35 299–305 10.1016/S0531-5565(00)00084-X 10832051

[B76] Mihaylova M. M. Shaw R. J. (2011). The AMPK signalling pathway coordinates cell growth, autophagy and metabolism. *Nat. Cell Biol.* 13 1016–1023 10.1038/ncb2329 21892142 PMC3249400

[B77] Min S. W. Cho S. H. Zhou Y. Schroeder S. Haroutunian V. Seeley W. W. (2010). Acetylation of tau inhibits its degradation and contributes to tauopathy. *Neuron* 67 953–966 10.1016/j.neuron.2010.08.044 20869593 PMC3035103

[B78] Morris K. C. Lin H. W. Thompson J. W. Perez-Pinzon M. A. (2011). Pathways for ischemic cytoprotection: role of sirtuins in caloric restriction, resveratrol, and ischemic preconditioning. *J. Cereb. Blood Flow Metab.* 31 1003–1019 10.1038/jcbfm.2010.229 21224864 PMC3070983

[B79] Navarro A. Boveris A. (2007). The mitochondrial energy transduction system and the aging process. *Am. J. Physiol. Cell Physiol.* 292 C670–C686 10.1152/ajpcell.00213.2006 17020935

[B80] Nemoto S. Fergusson M. M. Finkel T. (2005). SIRT1 functionally interacts with the metabolic regulator and transcriptional coactivator PGC-1{alpha}. *J. Biol. Chem.* 280 16456–16460 10.1074/jbc.M501485200 15716268

[B81] Nisoli E. Tonello C. Cardile A. Cozzi V. Bracale R. Tedesco L. (2005). Calorie restriction promotes mitochondrial biogenesis by inducing the expression of eNOS. *Science* 310 314–317 10.1126/science.1117728 16224023

[B82] Okawara M. Katsuki H. Kurimoto E. Shibata H. Kume T. Akaike A. (2007). Resveratrol protects dopaminergic neurons in midbrain slice culture from multiple insults. *Biochem. Pharmacol.* 73 550–560 10.1016/j.bcp.2006.11.003 17147953

[B83] Onyango P. Celic I. McCaffery J. M. Boeke J. D. Feinberg A. P. (2002). SIRT3, a human SIR2 homologue, is an NAD-dependent deacetylase localized to mitochondria. *Proc. Natl. Acad. Sci. U.S.A.* 99 13653–13658 10.1073/pnas.222538099 12374852 PMC129731

[B84] Palacios O. M. Carmona J. J. Michan S. Chen K. Y. Manabe Y. Ward J. L. III (2009). Diet and exercise signals regulate SIRT3 and activate AMPK and PGC-1alpha in skeletal muscle. *Aging (Albany NY)* 1 771–783 20157566 10.18632/aging.100075 PMC2815736

[B85] Pallas M. Pizarro J. G. Gutierrez-Cuesta J. Crespo-Biel N. Alvira D. Tajes M. (2008). Modulation of SIRT1 expression in different neurodegenerative models and human pathologies. *Neuroscience* 154 1388–1397 10.1016/j.neuroscience.2008.04.065 18538940

[B86] Pallos J. Bodai L. Lukacsovich T. Purcell J. M. Steffan J. S. Thompson L. M. (2008). Inhibition of specific HDACs and sirtuins suppresses pathogenesis in a *Drosophila* model of Huntington’s disease. *Hum. Mol. Genet.* 17 3767–3775 10.1093/hmg/ddn273 18762557 PMC2581431

[B87] Parker J. A. Arango M. Abderrahmane S. Lambert E. Tourette C. Catoire H. (2005). Resveratrol rescues mutant polyglutamine cytotoxicity in nematode and mammalian neurons. *Nat. Genet.* 37 349–350 10.1038/ng1534 15793589

[B88] Patel N. V. Gordon M. N. Connor K. E. Good R. A. Engelman R. W. Mason J. (2005). Caloric restriction attenuates Abeta-deposition in Alzheimer transgenic models. *Neurobiol. Aging*. 26 995–1000 10.1016/j.neurobiolaging.2004.09.014 15748777

[B89] Perry V. H. Lunn E. R. Brown M. C. Cahusac S. Gordon S. (1990). Evidence that the Rate of Wallerian degeneration is controlled by a single autosomal dominant gene. *Eur. J. Neurosci.* 2 408–413 10.1111/j.1460-9568.1990.tb00433.x 12106028

[B90] Petersen K. F. Befroy D. Dufour S. Dziura J. Ariyan C. Rothman D. L. (2003). Mitochondrial dysfunction in the elderly: possible role in insulin resistance. *Science* 300 1140–1142 10.1126/science.1082889 12750520 PMC3004429

[B91] Picard F. Kurtev M. Chung N. Topark-Ngarm A. Senawong T. Machado De Oliveira R. (2004). Sirt1 promotes fat mobilization in white adipocytes by repressing PPAR-gamma. *Nature* 429 771–776 10.1038/nature02583 15175761 PMC2820247

[B92] Puigserver P. Wu Z. Park C. W. Graves R. Wright M. Spiegelman B. M. (1998). A cold-inducible coactivator of nuclear receptors linked to adaptive thermogenesis. *Cell* 92 829–839 10.1016/S0092-8674(00)81410-5 9529258

[B93] Qin W. Chachich M. Lane M. Roth G. Bryant M. de Cabo R. (2006a). Calorie restriction attenuates Alzheimer’s disease type brain amyloidosis in Squirrel monkeys (*Saimiri sciureus*). *J. Alzheimers Dis.* 10 417–422 17183154 10.3233/jad-2006-10411

[B94] Qin W. Yang T. Ho L. Zhao Z. Wang J. Chen L. (2006b). Neuronal SIRT1 activation as a novel mechanism underlying the prevention of Alzheimer disease amyloid neuropathology by calorie restriction. *J. Biol. Chem.* 281 21745–21754 10.1074/jbc.M602909200 16751189

[B95] Qiu X. Brown K. Hirschey M. D. Verdin E. Chen D. (2010). Calorie restriction reduces oxidative stress by SIRT3-mediated SOD2 activation. *Cell Metab.* 12 662–667 10.1016/j.cmet.2010.11.015 21109198

[B96] Ramadori G. Lee C. E. Bookout A. L. Lee S. Williams K. W. Anderson J. (2008). Brain SIRT1: anatomical distribution and regulation by energy availability. *J. Neurosci.* 28 9989–9996 10.1523/JNEUROSCI.3257-08.2008 18829956 PMC2578850

[B97] Raval A. P. Dave K. R. Perez-Pinzon M. A. (2006). Resveratrol mimics ischemic preconditioning in the brain. *J. Cereb. Blood Flow Metab.* 26 1141–1147 10.1038/sj.jcbfm.9600262 16395277

[B98] Rodgers J. T. Lerin C. Haas W. Gygi S. P. Spiegelman B. M. Puigserver P. (2005). Nutrient control of glucose homeostasis through a complex of PGC-1alpha and SIRT1. *Nature* 434 113–118 10.1038/nature03354 15744310

[B99] Rogina B. Helfand S. L. (2004). Sir2 mediates longevity in the fly through a pathway related to calorie restriction. *Proc. Natl. Acad. Sci. U.S.A.* 101 15998–16003 10.1073/pnas.0404184101 15520384 PMC528752

[B100] Rose G. Dato S. Altomare K. Bellizzi D. Garasto S. Greco V. (2003). Variability of the SIRT3 gene, human silent information regulator Sir2 homologue, and survivorship in the elderly. *Exp. Gerontol.* 38 1065–1070 10.1016/S0531-5565(03)00209-2 14580859

[B101] Rosen D. R. (1993). Mutations in Cu/Zn superoxide dismutase gene are associated with familial amyotrophic lateral sclerosis. *Nature* 364 362 10.1038/364362c0 8332197

[B102] Sakakibara I. Fujino T. Ishii M. Tanaka T. Shimosawa T. Miura S. (2009). Fasting-induced hypothermia and reduced energy production in mice lacking acetyl-CoA synthetase 2. *Cell Metab.* 9 191–202 10.1016/j.cmet.2008.12.008 19187775

[B103] Salminen A. Kaarniranta K. (2012). AMP-activated protein kinase (AMPK) controls the aging process via an integrated signaling network. *Ageing Res. Rev.* 11 230–241 10.1016/j.arr.2011.12.005 22186033

[B104] Salminen A. Kauppinen A. Suuronen T. Kaarniranta K. (2008a). SIRT1 longevity factor suppresses NF-kappaB-driven immune responses: regulation of aging via NF-kappaB acetylation? *Bioessays* 30 939–942 10.1002/bies.20799 18800364

[B105] Salminen A. Ojala J. Huuskonen J. Kauppinen A. Suuronen T. Kaarniranta K. (2008b). Interaction of aging-associated signaling cascades: inhibition of NF-kappaB signaling by longevity factors FoxOs and SIRT1. *Cell. Mol. Life Sci.* 65 1049–1058 10.1007/s00018-008-7461-3 18193389 PMC11131652

[B106] Sasaki Y. Vohra B. P. Baloh R. H. Milbrandt J. (2009). Transgenic mice expressing the Nmnat1 protein manifest robust delay in axonal degeneration in vivo. *J. Neurosci.* 29 6526–6534 10.1523/JNEUROSCI.1429-09.2009 19458223 PMC2697066

[B107] Satoh A. Brace C. S. Ben-Josef G. West T. Wozniak D. F. Holtzman D. M. (2010). SIRT1 promotes the central adaptive response to diet restriction through activation of the dorsomedial and lateral nuclei of the hypothalamus. *J. Neurosci.* 30 10220–10232 10.1523/JNEUROSCI.1385-10.2010 20668205 PMC2922851

[B108] Sauve A. A. (2009). Pharmaceutical strategies for activating sirtuins. *Curr. Pharm. Des.* 15 45–56 10.2174/138161209787185797 19149602

[B109] Sauve A. A. Wolberger C. Schramm V. L. Boeke J. D. (2006). The biochemistry of sirtuins. *Annu. Rev. Biochem.* 75 435–465 10.1146/annurev.biochem.74.082803.133500 16756498

[B110] Schlicker C. Gertz M. Papatheodorou P. Kachholz B. Becker C. F. Steegborn C. (2008). Substrates and regulation mechanisms for the human mitochondrial sirtuins Sirt3 and Sirt5. *J. Mol. Biol.* 382 790–801 10.1016/j.jmb.2008.07.048 18680753

[B111] Schmitz M. L. Mattioli I. Buss H. Kracht M. (2004). NF-kappaB: a multifaceted transcription factor regulated at several levels. *Chembiochem* 5 1348–1358 10.1002/cbic.200400144 15457532

[B112] Schwer B. Bunkenborg J. Verdin R. O. Andersen J. S. Verdin E. (2006). Reversible lysine acetylation controls the activity of the mitochondrial enzyme acetyl-CoA synthetase 2. *Proc. Natl. Acad. Sci. U.S.A.* 103 10224–10229 10.1073/pnas.0603968103 16788062 PMC1502439

[B113] Schwer B. Eckersdorff M. Li Y. Silva J. C. Fermin D. Kurtev M. V. (2009). Calorie restriction alters mitochondrial protein acetylation. *Aging Cell* 8 604–606 10.1111/j.1474-9726.2009.00503.x 19594485 PMC2752488

[B114] Schwer B. North B. J. Frye R. A. Ott M. Verdin E. (2002). The human silent information regulator (Sir)2 homologue hSIRT3 is a mitochondrial nicotinamide adenine dinucleotide-dependent deacetylase. *J. Cell Biol.* 158 647–657 10.1083/jcb.200205057 12186850 PMC2174009

[B115] Shi T. Wang F. Stieren E. Tong Q. (2005). SIRT3, a mitochondrial sirtuin deacetylase, regulates mitochondrial function and thermogenesis in brown adipocytes. *J. Biol. Chem.* 280 13560–13567 10.1074/jbc.M414670200 15653680

[B116] Shimazu T. Hirschey M. D. Huang J. Y. Ho L. T. Verdin E. (2010). Acetate metabolism and aging: an emerging connection. *Mech. Ageing Dev.* 131 511–516 10.1016/j.mad.2010.05.001 20478325

[B117] Shimokawa I. Trindade L. S. (2010). Dietary restriction and aging in rodents: a current view on its molecular mechanisms. *Aging Dis.* 1 89–107 22396859 PMC3295025

[B118] Shindler K. S. Ventura E. Dutt M. Elliott P. Fitzgerald D. C. Rostami A. (2010). Oral resveratrol reduces neuronal damage in a model of multiple sclerosis. *J. Neuroophthalmol.* 30 328–339 10.1097/WNO.0b013e3181f7f833 21107122 PMC3312784

[B119] Shindler K. S. Ventura E. Rex T. S. Elliott P. Rostami A. (2007). SIRT1 activation confers neuroprotection in experimental optic neuritis. *Invest. Ophthalmol. Vis. Sci.* 48 3602–3609 10.1167/iovs.07-0131 17652729 PMC1964753

[B120] Shulga N. Wilson-Smith R. Pastorino J. G. (2010). Sirtuin-3 deacetylation of cyclophilin D induces dissociation of hexokinase II from the mitochondria. *J. Cell Sci.* 123 894–902 10.1242/jcs.061846 20159966 PMC3189253

[B121] Sinclair D. A. (2002). Paradigms and pitfalls of yeast longevity research. *Mech. Ageing Dev.* 123 857–867 10.1016/S0047-6374(02)00023-4 12044934

[B122] Singh K. K. (2004). Mitochondrial dysfunction is a common phenotype in aging and cancer. *Ann. N. Y. Acad. Sci.* 1019 260–264 10.1196/annals.1297.043 15247025

[B123] Someya S. Yu W. Hallows W. C. Xu J. Vann J. M. Leeuwenburgh C. (2010). Sirt3 mediates reduction of oxidative damage and prevention of age-related hearing loss under caloric restriction. *Cell* 143 802–812 10.1016/j.cell.2010.10.002 21094524 PMC3018849

[B124] Swerdlow R. H. (2007). Treating neurodegeneration by modifying mitochondria: potential solutions to a “complex” problem. *Antioxid. Redox Signal.* 9 1591–1603 10.1089/ars.2007.1676 17663643

[B125] Swerdlow R. H. (2011). Brain aging, Alzheimer’s disease, and mitochondria. *Biochim. Biophys. Acta* 1812 1630–1639 10.1016/j.bbadis.2011.08.012 21920438 PMC3210037

[B126] Tang B. L. (2009). Sirt1’s complex roles in neuroprotection. *Cell. Mol. Neurobiol.* 29 1093–1103 10.1007/s10571-009-9414-2 19462229 PMC11506029

[B127] Tao R. Coleman M. C. Pennington J. D. Ozden O. Park S. H. Jiang H. (2010). Sirt3-mediated deacetylation of evolutionarily conserved lysine 122 regulates MnSOD activity in response to stress. *Mol. Cell.* 40 893–904 10.1016/j.molcel.2010.12.013 21172655 PMC3266626

[B128] Tissenbaum H. A. Guarente L. (2001). Increased dosage of a sir-2 gene extends lifespan in *Caenorhabditis elegans*. *Nature* 410 227–230 10.1038/35065638 11242085

[B129] Viswanathan M. Guarente L. (2011). Regulation of *Caenorhabditis elegans* lifespan by sir-2.1 transgenes. *Nature* 477 E1–E2 10.1038/nature10440 21938026

[B130] Wakeling L. A. Ions L. J. Ford D. (2009). Could Sirt1-mediated epigenetic effects contribute to the longevity response to dietary restriction and be mimicked by other dietary interventions? *Age (Dordr.)* 31 327–341 10.1007/s11357-009-9104-5 19568959 PMC2813047

[B131] Wang J. Fivecoat H. Ho L. Pan Y. Ling E. Pasinetti G. M. (2010). The role of Sirt1: at the crossroad between promotion of longevity and protection against Alzheimer’s disease neuropathology. *Biochim. Biophys. Acta* 1804 1690–1694 10.1016/j.bbapap.2009.11.015 19945548

[B132] Wang J. Ho L. Qin W. Rocher A. B. Seror I. Humala N. (2005a). Caloric restriction attenuates beta-amyloid neuropathology in a mouse model of Alzheimer’s disease. *FASEB J.* 19 659–661 10.1096/fj.04-3182fje 15650008

[B133] Wang J. Zhai Q. Chen Y. Lin E. Gu W. McBurney M. W. (2005b). A local mechanism mediates NAD-dependent protection of axon degeneration. *J. Cell Biol.* 170 349–355 10.1083/jcb.200504028 16043516 PMC2171458

[B134] Wang P. Xu T. Y. Guan Y. F. Tian W. W. Viollet B. Rui Y. C. (2011). Nicotinamide phosphoribosyltransferase protects against ischemic stroke through SIRT1-dependent adenosine monophosphate-activated kinase pathway. *Ann. Neurol.* 69 360–374 10.1002/ana.22236 21246601

[B135] Wang S. Xing Z. Vosler P. S. Yin H. Li W. Zhang F. (2008). Cellular NAD replenishment confers marked neuroprotection against ischemic cell death: role of enhanced DNA repair. *Stroke* 39 2587–2595 10.1161/STROKEAHA.107.509158 18617666 PMC2743302

[B136] Wareski P. Vaarmann A. Choubey V. Safiulina D. Liiv J. Kuum M. (2009). PGC-1{alpha} and PGC-1{beta} regulate mitochondrial density in neurons. *J. Biol. Chem.* 284 21379–21385 10.1074/jbc.M109.018911 19542216 PMC2755862

[B137] Wood J. G. Rogina B. Lavu S. Howitz K. Helfand S. L. Tatar M. (2004). Sirtuin activators mimic caloric restriction and delay ageing in metazoans. *Nature* 430 686–689 10.1038/nature02789 15254550

[B138] Xiong S. Salazar G. Patrushev N. Alexander R. W. (2011). FoxO1 mediates an autofeedback loop regulating SIRT1 expression. *J. Biol. Chem.* 286 5289–5299 10.1074/jbc.M110.163667 21149440 PMC3037641

[B139] Yang Y. Hubbard B. P. Sinclair D. A. Tong Q. (2010). Characterization of murine SIRT3 transcript variants and corresponding protein products. *J. Cell. Biochem.* 111 1051–1058 10.1002/jcb.22795 20677216 PMC3558747

[B140] Yang Y. H. Chen Y. H. Zhang C. Y. Nimmakayalu M. A. Ward D. C. Weissman S. (2000). Cloning and characterization of two mouse genes with homology to the yeast Sir2 gene. *Genomics* 69 355–369 10.1006/geno.2000.6360 11056054

[B141] Yeung F. Hoberg J. E. Ramsey C. S. Keller M. D. Jones D. R Frye R. A. (2004). Modulation of NF-kappaB-dependent transcription and cell survival by the SIRT1 deacetylase. *EMBO J.* 23 2369–2380 10.1038/sj.emboj.7600244 15152190 PMC423286

